# DNMT3B isoforms without catalytic activity stimulate gene body methylation as accessory proteins in somatic cells

**DOI:** 10.1038/ncomms11453

**Published:** 2016-04-28

**Authors:** Christopher E. Duymich, Jessica Charlet, Xiaojing Yang, Peter A. Jones, Gangning Liang

**Affiliations:** 1 Department of Urology, USC Norris Comprehensive Cancer Center, Keck School of Medicine, University of Southern California, Los Angeles, California 90089, USA

## Abstract

Promoter DNA methylation is a key epigenetic mechanism for stable gene silencing, but is correlated with expression when located in gene bodies. Maintenance and *de novo* DNA methylation by catalytically active DNA methyltransferases (DNMT1 and DNMT3A/B) require accessory proteins such as UHRF1 and DNMT3L. DNMT3B isoforms are widely expressed, although some do not have active catalytic domains and their expression can be altered during cell development and tumourigenesis, questioning their biological roles. Here, we show that DNMT3B isoforms stimulate gene body methylation and re-methylation after methylation-inhibitor treatment. This occurs independently of the isoforms' catalytic activity, demonstrating a similar functional role to the accessory protein DNMT3L, which is only expressed in undifferentiated cells and recruits DNMT3A to initiate DNA methylation. This unexpected role for DNMT3B suggests that it might substitute for the absent accessory protein DNMT3L to recruit DNMT3A in somatic cells.

## Abstract (web summary)

*De novo* DNA methylation is carried out by DNA methyltransferase DNMT3A/B, although DNMT3B isoforms without active catalytic domains are widely expressed. Here, the authors show that DNMT3B isoforms stimulate gene body methylation and re-methylation independently of the isoforms' catalytic activity.

## Graphical abstract

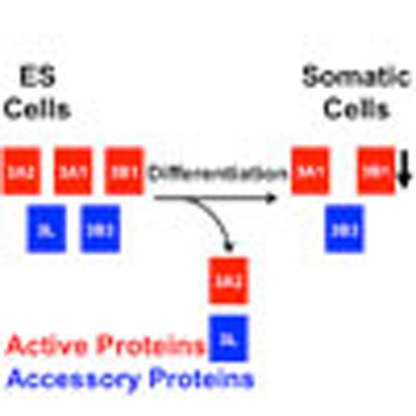

DNA methylation is a key epigenetic mechanism that participates in stable gene silencing in key biological processes, including the establishment and maintenance of tissue specific gene-expression patterns, X-chromosome inactivation, parasitic transposable elements silencing and genomic imprinting^1^,^2^,^3^,^4^. Although DNA methylation is critical for mammalian development, genome-wide studies show that aberrations of normal tissue DNA-methylation patterns are a hallmark of cancer and other diseases^5^,^6^.

Cytosines in a CpG context are methylated by the transfer of a methyl group from S-adenosylmethionine catalysed by DNA methyltransferases (DNMTs)^7^. DNMTs in mammals are comprised of four family members: DNMT1, DNMT3A, DNMT3B as well as DNMT3L (DNMT 3-like), which is required for the establishment of DNA-methylation patterns during development^8^. Maintenance of DNA methylation is carried out by DNMT1, which copies DNA-methylation patterns from the parental to the daughter strand during replication^9^. Both DNMT3A and DNMT3B serve as *de novo* methyltransferases during embryonic development but they also help maintain the DNA-methylation patterns in somatic cells in concert with DNMT1, since the latter cannot perform this function alone^10^,^11^,^12^. DNMT3A has 2 different isoforms, while DNMT3B has more than 30 isoforms^13^,^14^,^15^,^16^. Expression patterns of the latter are highly conserved between humans and mice, suggesting that these isoforms are biologically relevant^17^. Many studies have identified the specific roles of DNMTs in DNA methylation during development but the role of aberrant expression levels of DNMTs and isoforms, especially DNMT3B in cancer, leading to global DNA-methylation changes, is still unclear^11^,^13^,^14^,^15^,^16^,^18^,^19^,^20^,^21^,^22^.

Several studies have investigated the roles of DNMTs in more detail, demonstrating that DNMT3B participates in gene body methylation or re-methylation by targeting the H3K36me3 modification^4^,^23^,^24^,^25^,^26^,^27^,^28^. Disruption of the catalytic domains of all three DNMTs was recently characterized in human ES cells by CRISPR/Cas9 genome editing^24^. However, the disruption of DNMT3B1 left a truncated version very similar to the DNMT3B3 isoform in these cells^24^. Furthermore, this study also found that although DNMT3B1 is highly expressed in ES cells, DNMT3B1 expression decreases and DNMT3B3, which has a disrupted catalytic domain, becomes the dominant isoform expressed in somatic cells^24^. Thus the role of DNMT3B isoforms in ES and somatic cells is not well-characterized.

In this study, we sought to identify target sites of DNMT3B isoforms on a genome-wide level and their functional roles by characterizing a representative panel of DNMT3B isoforms and DNMT3L by restoring their expression in DNMT3B-deficient cells.

We confirm that transcribed regions of genes are the favoured *de novo* DNA-methylation target. We also show that isoforms of DNMT3B can influence DNA methylation in cells with decreased methylation and re-methylation in gene bodies after DNA-methylation-inhibitor treatment. Altogether, our results suggest that DNMT3B isoforms can act as accessory proteins that interact with catalytically active enzymes to re-establish DNA methylation and could be one of many key factors for initiation of *de novo* DNA methylation during tumourigenesis.

## Results

### Stable Reintroduction of DNMT Isoforms

**Figure 1:**
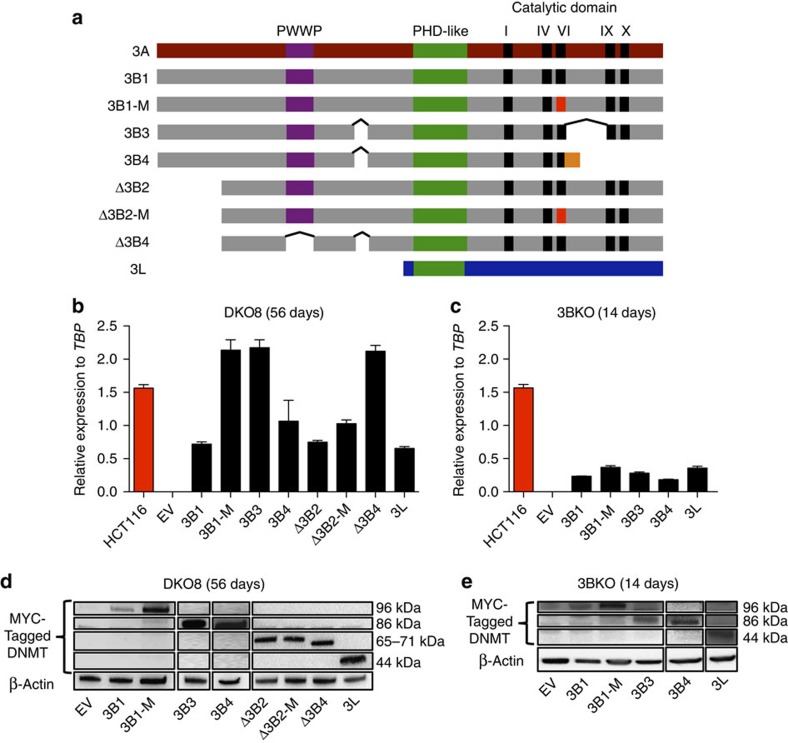
Stable reintroductions of DNMT3B isoforms and DNMT3L in DKO8 and 3BKO cell lines. (**a**) Schematic diagram of DNMT3A (3A), DNMT3B isoforms (3B1, 3B1-M, 3B3, 3B4, Δ3B2, Δ3B2-M and Δ3B4) and DNMT3L (3L) showing conserved PWWP (purple), PHD-like domain (green) and DNMT catalytic motifs (black). There are five catalytic domains (I, IV, VI, IX and X) in DNMT3A and DNMT3B, all of which are absent in DNMT3L. A red-coloured VI domain indicates inactivating mutations (Cys to Ser) of amino acids 651 and 452 in 3B1-M and Δ3B2-M, respectively. 3B4 has a frameshift with a unique protein sequence shown in orange. ^indicates alternative splicing. (**b**,**c**) mRNA expression level of endogenous *DNMT3B* in HCT116 cells, exogenous *DNMT3B* isoforms and *DNMT3L* assessed by qRT-PCR and normalized to the expression of the TATA Box Binding Protein (*TBP*) in DKO8 cells, 56 days post transfection and 3BKO cells, 14 days post transfection, respectively. Error bars indicate standard deviation from the mean of three biological replicates. The empty vector (EV) cell line is the transfection control. (**d**,**e**) Protein-expression levels of exogenous MYC-tagged DNMT isoforms by western blot analysis in DKO8 cells, 56 days post transfection and 3BKO cells, 14 days post transfection, respectively. β-ACTIN was used as a loading control.

We elucidated the role of DNMT3B and its isoforms in DNA methylation by using the 3BKO and DKO8 derivatives of the HCT116 colon cancer cell line that have homozygous deletions for *DNMT3B.* DKO8 cells additionally have a markedly reduced protein level of a hypomorphic DNMT1 (*DNMT1*^*ΔE2-5*^) (refs 29, 30) allele. There is globally about 3% less DNA methylation in 3BKO cells and 22% less DNA methylation in DKO8 cells compared with the parental line HCT116 (Supplementary Fig. 1)^29^. We selected a representative panel of DNMT3B isoforms, as well as DNMT3L, which is catalytically inactive and expressed in ES cells but not in somatic cells^8^,^31^, to use as a positive accessory protein control (Fig. 1a). For DNMT3B isoforms, we chose DNMT3B1: the canonical full-length protein, DNMT3B3: a catalytically inactive isoform^32^ expressed in somatic and ES cells but overexpressed in some types of cancer^33^, DNMT3B4: a catalytically inactive isoform that interacts with DNMT3s and reduces DNA-binding ability^32^, DNMTΔ3B2: a N-terminal-truncated isoform mainly overexpressed in lung cancer^15^, DNMTΔ3B4: a N-terminal-truncated isoform missing the PWWP domain^15^ and DNMT3L: expressed only during embryonic development^34^. In addition, we engineered DNMT3B1-M and DNMTΔ3B2-M, which are catalytically inactive mutants containing a cysteine to serine inactivating point mutation at position 651 and 452, respectively^35^. After infection we obtained polyclonal cell populations expressing a specific DNMT3B isoform or DNMT3L in DKO8 and 3BKO cells.

**Figure 2:**
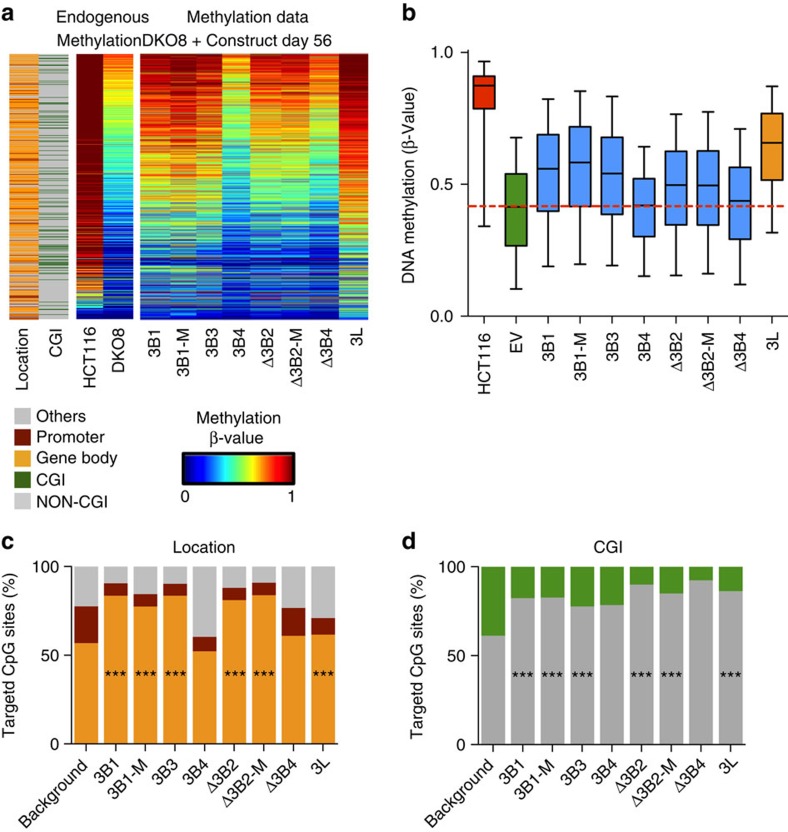
DNMT3B isoforms and DNMT3L restore DNA methylation at specific CpG sites. (**a**) Heatmap showing 54,911 CpG sites in DKO8 cells, expressing the indicated DNMT isoforms. CpGs were selected because they were targeted by at least one construct. CpG sites in genomic locations targeted by DNMT3Bs and 3L with respect to promoter (maroon), gene body (orange) and other regions, excluding promoters and gene bodies (grey), are shown in the left panel. CpG sites in CpG islands are represented in green in the left panel. Endogenous methylation levels in HCT116 and DKO8 cells are represented by a cold to warm colour scale (*β*-value 0–1, 0–100% methylated), where every row represents one CpG. DNA-methylation levels of DKO8 cells expressing a DNMT3B isoforms or DNMT3L are shown in the right panel. (**b**) Boxplots showing the distribution of DNA-methylation levels of 54,911 CpG sites for each indicated cell line. HCT116 cells' methylation level is included for comparison with the derivative cell line DKO8 EV. A dashed red line indicates the median of DKO8 EV, outliers outside the 5th to 95th percentiles are not shown. (**c**) Distribution of genomic locations targeted by individual DNMT3Bs and 3L with respect to promoter (maroon), gene body (orange) and other regions excluding promoters and gene bodies (grey), from the 450 K array. (**d**) Distribution of genomic locations targeted by DNMT3Bs and 33LL in respect to CpG island (green) or non-CpG island (grey) from the 450 K array. Background distribution is the representative population of CpG sites that could be targeted by the individual DNMTs. Statistical enrichment was performed using a *z*-test comparing the targeted CpG sites for each DNMT isoform with the overall distribution of probes on the 450 K array, ***P<0.001.

Different RNA-expression levels were seen for each of the *DNMT*s but larger variations were apparent at the protein levels, possibly reflecting differential stabilities of the variable isoforms outside of the putative DNMT3A/3B complex (Fig. 1b–e). Endogenous *DNMT3A* and exogenous *DNMT3B* or *DNMT3L* RNA-expression levels were measured by qRT-PCR, following puromycin selection in both cell lines and before and after 5-Aza-CdR treatment in 3BKO cells (Supplementary Fig. 2). Consistent with our previous study^35^, we show the mutant form of DNMT3B1 was expressed at a higher protein level than its wild-type form; however, no significant differences in restoration of DNA methylation between the transfected constructs could be observed (Fig. 2b). We also know that excess DNMT3B protein is degraded when not bound to nucleosomes, which may explain the differences between the RNA and protein levels of these DNMTs^35^.

### DNA-methylation restoration by DNMT3B isoforms

Global DNA-methylation levels in DKO8 cells expressing DNMT3B isoforms or DNMT3L were measured 56 days post transfection. The results are compared with the parental HCT116 cell line and represented as boxplots (Supplementary Fig. 3a). CpG sites that showed an increase of 0.2 in *β*-value, compared with the control EV with any construct, were retained for further analysis and the resulting methylation values are shown as heatmaps, boxplots (Fig. 2a,b) or *δ*-methylation values (Supplementary Fig. 3b). As expected, DNMT3L could restore DNA methylation in the transfected cells and showed the strongest overall restoration of DNA methylation compared with the DNMT3B isoforms (Fig. 2a). Interestingly, the majority of sites showing increased DNA methylation were also methylated in the parental HCT116 cells (Fig. 2a). Most of these restored sites are located in gene bodies, which were found to be enriched in H3K36me3 in HCT116 cells, a characteristic of an actively transcribed region^23^. We previously showed that the H3K36me3 mark is not lost in the more severely demethylated derivative cell line DKO1, which has lost 90% of DNA methylation^23^. This finding is consistent with other observations that DNMT3B recognizes the H3K36me3 modification written by SETD2 (refs 25, 26). Individually, the sites targeted by each DNMT3B isoform which showed a 0.2 increase in *β*-value compared with the control EV are located in gene bodies and non-CpG islands regardless of the isoform when compared with the background distribution of CpG sites on the array (Fig. 2a,c,d) except for DNMT3B4 and DNMTΔ3B4. Biological replicates for 3 of the 9 DNMT constructs were performed, demonstrating that the CpG site-specific recruitment was consistent globally (Supplementary Fig. 4) and for the specific target sites (Supplementary Figs 5–7).

Furthermore, DNMT3B1 and its mutated form DNMT3B1-M show similar restoration of DNA methylation regardless of the latter being catalytically inactive, consistent with previous findings (Fig. 2a,b)^35^. In addition, although DNMT3B3 has been considered as a catalytically inactive isoform^32^, DNA methylation was strongly increased by its presence. The catalytically active DNMTΔ3B2 and inactive Δ3B2-M isoforms both lack the N-terminal domain and have similar decreased abilities to methylate DNA (Fig. 2a,b). DNMTΔ3B4 that has no PWWP domain had the lowest ability to induce re-methylation. DNMT3B4 was also weak at inducing re-methylation, confirming the finding of its decreased binding affinity with DNA^32^.

The abilities of DNMT3B and its isoforms to restore methylation are mainly dependent on the presence of an N-terminal but not a catalytic domain in most of the isoforms (Fig. 2a,b). The exception here is DNMT3B4, which loses the ability to restore DNA methylation and has previously been shown to cause hypomethylation by binding to active DNMT3s and reducing their DNA-binding affinities^20^,^32^. The loss of DNA or nucleosome-binding affinity of DNMT3B4 could be due to the specific truncated C-terminal structure of DNMT3B4, which should be further studied. However, our previous studies have demonstrated that the ability of DNMT3B1 to bind nucleosomes is important in maintaining DNA-methylation levels, while loss of the N-terminal domain could markedly decrease the binding ability and increase protein degradation^35^,^36^. Unexpectedly, most DNMT3B isoforms can restore DNA methylation at similar target regions independently of the presence of functional catalytic domains. These findings suggest that besides its functional role as a methyltransferase, DNMT3B may also act as an accessory protein to recruit DNMT3A and stimulate DNA methylation.

### Re-methylation rate increased by DNMT3B isoforms

We recently showed that inhibiting gene body methylation by transient 5-Aza-CdR treatment decreases the expression of some genes, and that restoration of this methylation requires DNMT3B (ref. 23). We demonstrated that there are four groups (I–IV) of demethylated targets, based on the rate of re-methylation in HCT116 cells after treatment with 5-Aza-CdR (Supplementary Fig. 8a)^23^. The CpG sites of Group I were enriched in gene bodies having the H3K36me3 modifications and showed the fastest rates of re-methylation and were almost fully remethylated 42 days after treatment. The CpG sites in Group IV, however, showed the slowest rate of re-methylation and remained demethylated at day 42 (ref. 23).

**Figure 3:**
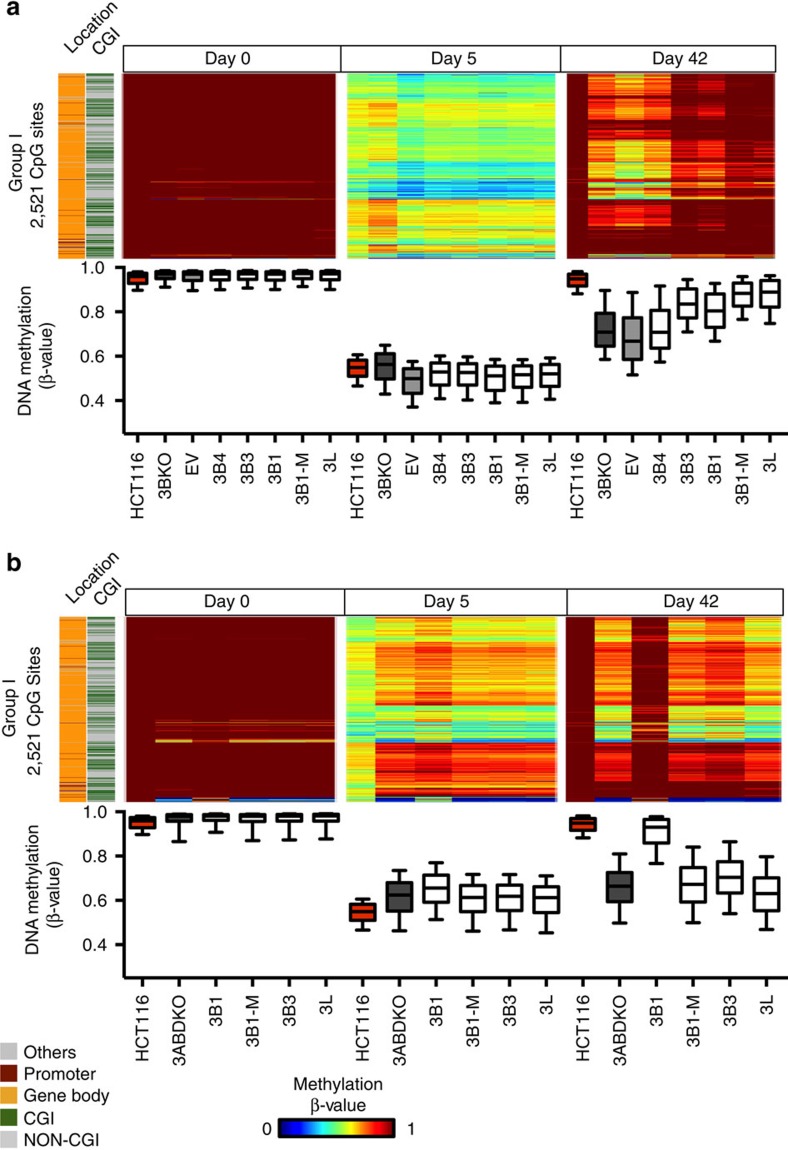
DNMT3B isoforms restore DNA methylation in a DNMT3B knock-out cell line. Heatmaps and Boxplots showing previously (Yang *et al*.^23^) defined H3K36me3-enriched and fast-rebounding Group I CpG sites in cell lines expressing different DNMTs before 24 h 5-Aza-CdR treatment and at Day 5 and Day 42 post treatment for 3BKO (**a**) and 3ABDKO (**b**) cells. Individual CpG sites falling in genomic locations targeted by DNMT3Bs and 3L with respect to promoter (maroon), gene body (orange) and other regions, excluding promoters and gene bodies (grey) are shown in the left panel. CpG sites in CpG islands are represented in green in the left panel. Endogenous methylation levels in cell lines are represented by a cold to warm colour scale (*β*-value 0–1, 0–100% methylation) where every row represents one CpG site in the three right panels.

To test whether DNMT3B isoforms have different functional roles during re-methylation, we stably introduced a subset of isoforms into the 3BKO cell line (Fig. 1a,c,e) and subsequently treated them with 5-Aza-CdR for 24 h. We observed DNA methylation decreases 5 days post treatment and DNA-methylation restoration 42 days post treatment, consistent with pervious studies (Fig. 3 and Supplementary Fig. 8)^23^. RNA-expression levels of endogenous *DNMT3A* and the exogenous *DNMT3B* or *DNMT3L* were analysed before and after 5-Aza-CdR treatment (Supplementary Fig. 2). Interestingly, *DNMT3B4* and *DNMT3L* showed decreased RNA-expression levels after 5-Aza-CdR treatment, although DNMT3L was still able to stimulate the DNA-methylation levels after 42 days (Fig. 3 and Supplementary Fig. 2b). DNMT3L, DNMT3B1, 3B1-M and 3B3 were able to stimulate DNA re-methylation most strongly at the H3K36me3-enriched Group I CpGs (Fig. 3)^23^. DNMT3B4 did not influence the rebound methylation of this group as we expected, since it has decreased binding affinity to DNA^32^. As we predicted, Group IV CpG sites were also confirmed to be non-DNMT3B target regions, the same as for DNMT3L (Supplementary Fig. 8).

To confirm that DNMT3A is actually the enzyme responsible for the DNA-methylation rebound, we used another HCT116 derivative cell line, 3ABDKO, which has homozygous deletions of *DNMT3A* and *DNMT3B* in addition to a single allele of *DNMT1* (ref. 37). We stably introduced a subset of isoforms into the 3ABDKO cell line and subsequently treated them with 5-Aza-CdR for 24 h. In general, these isoforms are stably expressed up to 42 days after treatment (Supplementary Fig. 9). We observed that only DNMT3B1 could robustly restore DNA methylation, while the catalytically inactive forms, mutant DNMT3B1, DNMT3B3 and DNMT3L, did not elicit the re-methylation at a majority of targets in the absence of DNMT3A (Fig. 3b). Although methylation was restored at a small portion of the targets in cells with or without isoforms of DNMT3B or DNMT3L (Fig. 3b), this could be explained by the known *de novo* activity of DNMT1 at specific regions^10^,^37^. In addition, 86% of these sites also gained DNA methylation in the 3BKO cell lines (Fig. 3a). This result therefore not only confirmed the well-known functional role of DNMT3B1 that is mainly expressed in ES cells^24^, but also a completely novel role as accessory protein for DNMT3B especially the catalytically inactive DNMT3B isoforms, such as DNMT3B3, which is highly expressed in somatic cells^24^. As we expected, Group IV CpGs were not showing increases in DNA methylation (Supplementary Fig. 10). These results therefore confirm that DNMT3B can act as an accessory protein like DNMT3L to establish DNA methylation by recruiting DNMT3A in somatic cells.

### Overexpression of DNMT3s in different cancer types

**Figure 4:**
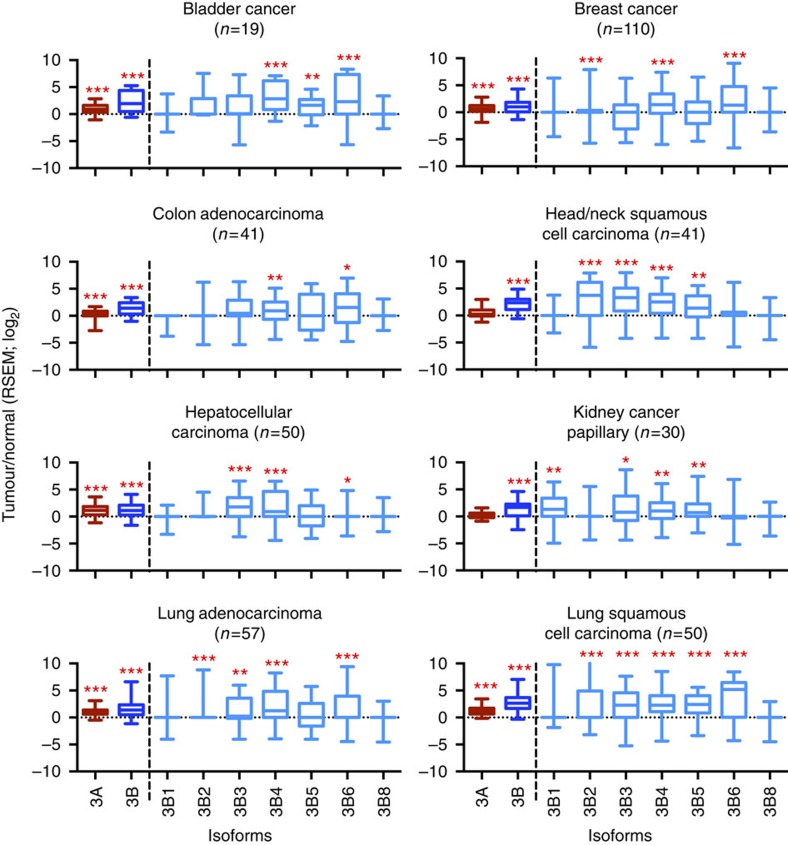
Differential expression levels of *DNMT3A* and *3B* between normal and various tumour tissues. Normalized read counts from RNA-Seq data were calculated for DNMT3A, DNMT3B and isoforms from matched normal and tumour tissue samples by expectation-maximization (RSEM). Expression fold change is shown as log_2_ (tumour/normal) for each cancer type. N indicates the number of sample pairs for each tumour type. Mann–Whitney's unpaired statistical test was used to assess expression differences on log_2_ (RSEM) values between tumour and normal tissue. *P<0.05, **P<0.01 or ***P<0.001.

The role of DNMT3B in acting as an accessory protein may also contribute to aberrant *de novo* methylation during tumourigenesis. Since overexpression of DNMTs^38^, especially DNMT3s and their isoforms, is commonly seen in tumours^12^,^19^,^29^,^39^. Our previous work suggests that DNMT3A requires DNMT3B for restoration of methylation in somatic cells and DNMT3B is also required for re-methylation after 5-Aza-CdR treatment^23^,^35^; however, the functional role of DNMT3B and its isoforms in a complex with DNMT3A is still not clear. DNMTs are overexpressed in cancer cells^19^, although detailed analysis of the expression profiles has not been described. We took advantage of The Cancer Genome Atlas (TCGA) project, which includes RNA-seq data on eight primary tumour types, to study expression levels of DNMT3s in normal tissues and their corresponding tumours. We confirmed overexpression of DNMT3A in six out of eight cancer types and DNMT3B overexpression in all cancer types, compared with normal tissues (Fig. 4). In addition, multiple isoforms of DNMT3B were also significantly overexpressed in these types of cancers (Fig. 4), including catalytically inactive DNMT3B3. Delta isoforms, DNMTΔ3B2 and four were purposefully not included in this analysis because of the difficulty to distinguish these isoforms by RNA-seq data (Fig. 4).

## Discussion

In this study we investigated the DNA-methylation target sites of DNMT3B isoforms on a genome-wide level by restoring the expression of a representative panel of DNMT3B isoforms in two different DNMT3B-deficient cell lines. Genome-wide analysis of DNA-methylation changes reveals that catalytic activity is not required for the induced DNA methylation, indicating an accessory protein role for DNMT3B isoforms. The presence of the N-terminal and PWWP domains was found to be important for DNMT3B function and its role as an accessory protein, except for DNMT3B4. Preferential binding of DNMT3B isoforms to regions known to be enriched with the H3K36me3 modification was found in our earlier work, as well as in others^23^,^25^,^26^. In addition, we confirmed the aberrant expression of DNMT3B isoforms in various cancers, using publically available data sets. Taken altogether, we show that DNMT3B can act as an accessory protein to restore DNA methylation of gene bodies in differentiated cells.

Our results, and other studies, have shown that DNMT3Bs are able to bind to the active DNMT3A molecules *in vivo* and *in vitro*^32^. The data here clearly show that DNMT3B1 can act as an accessory protein even when it is catalytically inactive, although it seems to require intact N-terminal and PWWP domains with the exception of DNMT3B4, thus further studies are required. Since catalytically inactive DNMT3B1 can establish *de novo* DNA-methylation patterns in both DNMT1 and DNMT3B-deficient cells but not in DNMT3B and DNMT3A deficient cells, it seems most likely that DNMT3B acts as an accessory protein for DNMT3A rather than DNMT1 (ref. 35). Although DNMT3B3 catalytic activity is controversial, our study suggests that DNA methylation in the presence of the DNMT3B3 isoform could be caused by it acting as an accessory protein in differentiated cells. This interpretation is consistent with the results of Liao *et al*.^24^ which showed that DNMT3A was necessary for *de novo* methylation of gene bodies during ES cell differentiation.

**Figure 5:**
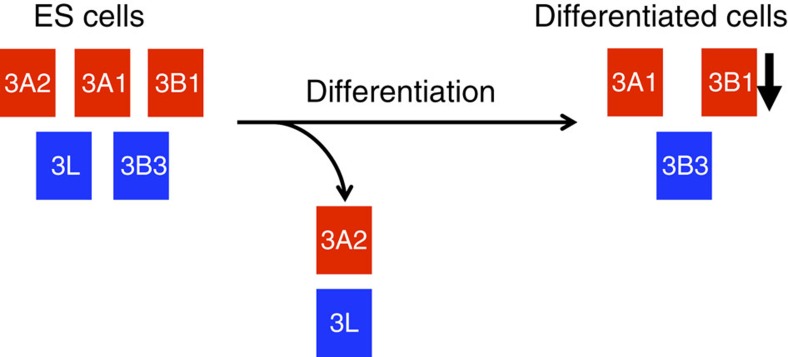
The changing landscape of *de novo* DNMTs during development. In ES cells the catalytically active CpG methyltransferases DNMT3A1 (3A1), DNMT3A2 (3A2) and DNMT3B1 (3B1) are expressed, as well as the accessory protein DNMT3L (3L) (refs 40, 44, 53, 54). Our work suggests that catalytically inactive DNMT3B3 (3B3) may also participate as an accessory protein in the establishment of DNA-methylation patterns. Following differentiation 3A2 and 3L are no longer expressed, while expression of 3B1 is decreased markedly, leaving 3A1 and the accessory protein 3B3 as potential mediators of methylation^53^,^54^,^55^. Our work also suggests that 3B3 preferentially targets 3A1 to gene bodies. Active methyltransferase enzymes and accessory proteins are shown in red and blue boxes, respectively.

The changing landscapes of catalytically active and inactive proteins included in ES and differentiated cells are depicted in Fig. 5. DNMT3L has been identified as a DNMT3A accessory protein to stimulate *de novo* methylation in ES cells^8^,^40^,^41^,^42^,^43^,^44^,^45^, while UHRF1 has also been recognized as a DNMT1 accessory protein to maintain DNA-methylation patterns in embryonic and differentiated cells^46^,^47^. Our findings, for the first time, suggest a role for DNMT3B as an accessory protein for DNMT3A to stimulate and restore DNA methylation. While DNMT3A alone cannot restore DNA methylation in 3BKO cells, DNMT3B1 can achieve this in 3ABDKO cells. This suggests that DNMT3B1 can have a dual role in acting as both a methyltransferase and an accessory protein. The latter role could be especially pronounced when different isoforms of DNMT3B are expressed simultaneously, interacting with each other to boost DNA methylation^32^. Furthermore, DNMT3L recruits DNMT3s to specifically bind to nucleosomes without H3K4 methylation and stimulate *de novo* methylation during embryonic development^34^,^41^. Our results showing overlapping target regions by DNMT3B and DNMT3L suggest that H3K36me3 could also be a target signal for DNMT3L but this requires further studies.

In conclusion, we have confirmed that DNMT3B isoforms can act as accessory proteins to restore DNA methylation, specifically in gene bodies in differentiated cells. This finding also suggests that DNMT3B and its catalytically inactive isoforms play key roles in collaborating with DNMT3A to initiate and maintain DNA methylation in transcribed regions. DNMT3A on the other hand has a low capability of restoring DNA methylation by itself without involvement of DNMT3B in differentiated cells. Our findings might also help explain the presence of aberrant DNA-methylation patterns during tumourigenesis due to the overexpression of DNMT3B isoforms, which could be a driving force for *de novo* methylation.

## Methods

### Cell lines and drug treatment

HCT116 derivative cell lines DKO8, 3BKO and 3ABDKO were obtained from Dr Baylin's laboratory and cultured in McCoy's 5A medium containing 1% penicillin/streptomycin and 10% inactivated fetal bovine serum in a humidified and 5% CO_2_ containing atmosphere at 37 **°**C. All cell lines were verified to be free of *Mycoplasma* contamination before use. Cell lines were treated with 0.3 μM of 5-Aza-CdR (Sigma-Aldrich) for 24 h followed by a medium change.

### DNMT isoform constructs

Human DNMT isoforms 3B1, Δ3B2, 3B3, Δ3B4 and 3L, containing a *MYC*-tagged DNA sequence ligated to the 5′- ends, were amplified from pIRESpuro/Myc constructs^48^ (a modified version of the pIRESpuro3 vector, Clontech), a gift from Dr Allen Yang (USC). Catalytically inactive mutants containing a cysteine to serine alteration in position 651 of DNMT3B1 and 452 of DNMTΔ3B2 proteins were established as previously described^35^. *MYC*-tagged DNMT sequences were cloned into pLJM1 lentivirus vector at *EcoRI* and *AgeI* sites using Infusion HD PCR Cloning Plus (Clontech) following the manufacturer's protocol. To produce lentivirus for the specific constructs, the vesicular stomatitis virus envelope protein G-expression construct pMD.G1, the packaging vector pCMV ΔR8.91 and transfer vector pLJM1 were used as previously described^49^. All vectors were amplified and purified using the PureYield Plasmid Maxiprep system (Promega), according to the manufacturer's instructions. The HCT116 derivative cell lines 3BKO, 3ABDKO and DKO8 were stably transfected with a lentivirus and selected with 2 μg ml^−1^ puromycin for 14, 14 and 21 days, respectively.

### Protein extraction and western blot analysis

Cells were trypsinised, washed with PBS and resuspended in RIPA buffer (50 mM Tris-HCl, ph 8.0, 150 mM NaCl, 1% NP-40, 0.5% DOC, 0.1% SDS) with protease inhibitors (Roche, 04693132001). The lysed cells were then sonicated on ice and cellular debris were removed by centrifugation. In all, 2 μg of protein was mixed with SDS/β-mercaptoethanol loading buffer and resolved on a BioRad 4-15% gradient SDS/PAGE gel. Antibodies against the MYC-epitope tag (Millipore, 05-724, 1:1,000) and β-ACTIN (Sigma, A2228, 1:2,000) as loading control were used. Proteins were visualized using the ECL detection system (Thermo Scientific) and BioRad's ChemiDoc system. Protein was extracted on day 56 for DKO8 cells and day 14 for 3BKO cells. The full-size images are shown in Supplementary Fig. 11.

### RNA isolation and qRT-PCR

Puromycin-resistant polyclonal cells, stably transfected with DNMT isoforms, were used for total RNA extraction using the RNeasy Mini kit (Qiagen). In all, 1** **μg of total RNA was treated with 10 U of DNaseI (Roche) at 25 °C for 20 min followed by 8 mM final concentration of EDTA and 10 min heat inactivation at 75 °C. Typically, 1 μg of DNase treated RNA was reverse transcribed using iScript Reverse Transcription Supermix (BioRad), according to the manufacturer's instructions. PCR reactions were performed using KAPA SYBR FAST University 2 × qPCR Master Mix. Primer sequences used in qRT-PCR: TBP sense 5′-GCCCGAAACGCCGAATAT-3′, antisense 5′-CCGTGGTTCGTGGCTCTCT-3′; PCNA sense 5′-GCAGATGTACCCCTTGTTGTAGAGT-3′, antisense 5′-TCTTCATCCTCGATCTTGGGA-3′; DNMT3A sense 5′-CAATGACCTCTCCATCGTCAAC-3′, antisense 5′-CATGCAGGAGGCGGTAGAA-3′; DNMT3B sense 5′-CTGCCGGTGTTTCTGTGTGG-3′, antisense 5′-TGTAACAGCTCCAGGGCTCC-3′; DNMT3L sense 5′-TACGCACGGCCCAAGC-3′, antisense 5′-ACCAGATTGTCCACGAACATCC-3′. RNA-expression analysis was done on days 21 and 56 post transfection on DKO8 cells. RNA-expression analysis was done on day 14 post transfection on 3BKO and 3ABDKO cells and on days 0 and 42 post 5-Aza-CdR treatment.

### Illumina Infinium HM450 DNA-methylation data processing

DNA was extracted after 56 days of DNMT re-expression. DNA methylation was assessed using Illumina's Infinium HumanMethylation450 (HM450) BeadChip array and was performed at the USC Epigenome Center according to the manufacturer's specifications. The array examines the DNA-methylation status of 482,421 CpG sites and each is reported as a *β*-value, ranging from 0 (unmethylated) to 1 (fully methylated). CpG probes with a detection *P* value >0.05, located within 15 base pairs of a single-nucleotide polymorphism or located in gene deserts, were excluded from further analysis in all samples leaving 385,826. Analysis and *β*-value calculations were performed as described elsewhere^23^,^50^,^51^. Changes of 0.2 (20%) *β*-value were considered target sites for analyses and are described elsewhere^48^. Statistical enrichment was performed using a *z*-test comparing the targeted CpG sites for each DNMT isoform with the overall distribution of probes on the 450 K array. DKO8 cells at day 56 were analysed for DNA methylation on the HM450 array, as well as 3BKO and 3 ABDKO cells on days 0, 5 and 42 post 5-Aza-CdR treatment.

### RNA-seq data collection and analysis

RNASeqV2 Level 3 data from TCGA was obtained from the publically available data portal (http://cancergenome.nih.gov/dataportal/). Values for gene-expression values were aligned using MapSplice and quantification was performed using RSEM^52^.

### Data availability

The genome-wide DNA-methylation data that support the findings of this study have been deposited in the Gene Expression Omnibus (GEO) database with the accession codes GSE51815 and GSE68344.

## Additional information

**Accession codes:** The DNA-methylation data obtained from the Illumina's Infinium HumanMethylation450 BeadChip have been deposited in the Gene Expression Omnibus database under the accession code GSE68344.

**How to cite this article:** Duymich, C. E. *et al*. DNMT3B Isoforms without catalytic activity stimulate gene body methylation as accessory proteins in somatic cells. *Nat. Commun.* 7:11453 doi: 10.1038/ncomms11453 (2016).

## Supplementary Material

Supplementary Information Supplementary Figures 1-11 and Supplementary Reference

## References

[b1] Bird A. DNA methylation patterns and epigenetic memory. Genes Dev. 16, 6–21 (2002). 11782440 10.1101/gad.947102

[b2] Jurkowska R. Z., Jurkowski T. P. & Jeltsch A. Structure and function of mammalian DNA methyltransferases. Chembiochem 12, 206–222 (2011). 21243710 10.1002/cbic.201000195

[b3] Esteller M. Cancer epigenetics for the 21st century: what's next? Genes Cancer 2, 604–606 (2011). 21941616 10.1177/1947601911423096 PMC3174266

[b4] Jones P. A. Functions of DNA methylation: islands, start sites, gene bodies and beyond. Nat. Rev. Genet. 13, 484–492 (2012). 22641018 10.1038/nrg3230

[b5] Jones P. A. & Baylin S. B. The epigenomics of cancer. Cell 128, 683–692 (2007). 17320506 10.1016/j.cell.2007.01.029 PMC3894624

[b6] Sharma S., Kelly T. K. & Jones P. A. Epigenetics in cancer. Carcinogenesis 31, 27–36 (2010). 19752007 10.1093/carcin/bgp220 PMC2802667

[b7] Goll M. G. & Bestor T. H. Eukaryotic cytosine methyltransferases. Annu. Rev. Biochem. 74, 481–514 (2005). 15952895 10.1146/annurev.biochem.74.010904.153721

[b8] Gowher H., Liebert K., Hermann A., Xu G. & Jeltsch A. Mechanism of stimulation of catalytic activity of Dnmt3A and Dnmt3B DNA-(cytosine-C5)-methyltransferases by Dnmt3L. J. Biol. Chem. 280, 13341–13348 (2005). 15671018 10.1074/jbc.M413412200

[b9] Chen T. . Complete inactivation of DNMT1 leads to mitotic catastrophe in human cancer cells. Nat. Genet. 39, 391–396 (2007). 17322882 10.1038/ng1982

[b10] Liang G. . Cooperativity between DNA methyltransferases in the maintenance methylation of repetitive elements. Mol. Cell. Biol. 22, 480–491 (2002). 11756544 10.1128/MCB.22.2.480-491.2002 PMC139739

[b11] Chen T., Ueda Y., Dodge J. E., Wang Z. & Li E. Establishment and maintenance of genomic methylation patterns in mouse embryonic stem cells by Dnmt3a and Dnmt3b. Mol. Cell. Biol. 23, 5594–5605 (2003). 12897133 10.1128/MCB.23.16.5594-5605.2003 PMC166327

[b12] Jones P. A. & Liang G. Rethinking how DNA methylation patterns are maintained. Nat. Rev. Genet. 10, 805–811 (2009). 19789556 10.1038/nrg2651 PMC2848124

[b13] Ostler K. R. . Cancer cells express aberrant DNMT3B transcripts encoding truncated proteins. Oncogene 26, 5553–5563 (2007). 17353906 10.1038/sj.onc.1210351 PMC2435620

[b14] Xie S. . Cloning, expression and chromosome locations of the human DNMT3 gene family. Gene 236, 87–95 (1999). 10433969 10.1016/s0378-1119(99)00252-8

[b15] Wang J., Walsh G., Liu D. D., Lee J. J. & Mao L. Expression of Delta DNMT3B variants and its association with promoter methylation of p16 and RASSF1A in primary non-small cell lung cancer. Cancer Res. 66, 8361–8366 (2006). 16951144 10.1158/0008-5472.CAN-06-2031

[b16] Gopalakrishnan S. . A novel DNMT3B splice variant expressed in tumor and pluripotent cells modulates genomic DNA methylation patterns and displays altered DNA binding. Mol. Cancer Res. 7, 1622–1634 (2009). 19825994 10.1158/1541-7786.MCR-09-0018 PMC2783805

[b17] Okano M., Xie S. & Li E. Cloning and characterization of a family of novel mammalian DNA (cytosine-5) methyltransferases. Nat. Genet. 19, 219–220 (1998). 9662389 10.1038/890

[b18] Okano M., Bell D. W., Haber D. A. & Li E. DNA methyltransferases Dnmt3a and Dnmt3b are essential for *de novo* methylation and mammalian development. Cell 99, 247–257 (1999). 10555141 10.1016/s0092-8674(00)81656-6

[b19] Robertson K. D. . The human DNA methyltransferases (DNMTs) 1, 3a and 3b: coordinate mRNA expression in normal tissues and overexpression in tumors. Nucleic Acids Res. 27, 2291–2298 (1999). 10325416 10.1093/nar/27.11.2291 PMC148793

[b20] Saito Y. . Overexpression of a splice variant of DNA methyltransferase 3b, DNMT3b4, associated with DNA hypomethylation on pericentromeric satellite regions during human hepatocarcinogenesis. Proc. Natl Acad. Sci. USA 99, 10060–10065 (2002). 12110732 10.1073/pnas.152121799 PMC126624

[b21] Varley K. E. . Dynamic DNA methylation across diverse human cell lines and tissues. Genome Res. 23, 555–567 (2013). 23325432 10.1101/gr.147942.112 PMC3589544

[b22] Kulis M. . Epigenomic analysis detects widespread gene-body DNA hypomethylation in chronic lymphocytic leukemia. Nat. Genet. 44, 1236–1242 (2012). 23064414 10.1038/ng.2443

[b23] Yang X. . Gene body methylation can alter gene expression and is a therapeutic target in cancer. Cancer Cell 26, 1–14 (2014). 25263941 10.1016/j.ccr.2014.07.028 PMC4224113

[b24] Liao J. . Targeted disruption of DNMT1, DNMT3A and DNMT3B in human embryonic stem cells. Nat. Genet. 47, 469–478 (2015). 25822089 10.1038/ng.3258 PMC4414868

[b25] Baubec T. . Genomic profiling of DNA methyltransferases reveals a role for DNMT3B in genic methylation. Nature 520, 243–247 (2015). 25607372 10.1038/nature14176

[b26] Morselli M. . In vivo targeting of *de novo* DNA methylation by histone modifications in yeast and mouse. Elife 4, e06205 (2015). 25848745 10.7554/eLife.06205 PMC4412109

[b27] Jones P. A., Rideout W. M., Shen J. C., Spruck C. H. & Tsai Y. C. Methylation, mutation and cancer. Bioessays 14, 33–36 (1992). 1546979 10.1002/bies.950140107

[b28] Maunakea A. K. . Conserved role of intragenic DNA methylation in regulating alternative promoters. Nature 466, 253–257 (2010). 20613842 10.1038/nature09165 PMC3998662

[b29] Rhee I. . DNMT1 and DNMT3b cooperate to silence genes in human cancer cells. Nature 416, 552–556 (2002). 11932749 10.1038/416552a

[b30] Egger G. . Identification of DNMT1 (DNA methyltransferase 1) hypomorphs in somatic knockouts suggests an essential role for DNMT1 in cell survival. Proc. Natl Acad. Sci. USA 103, 14080–14085 (2006). 16963560 10.1073/pnas.0604602103 PMC1599915

[b31] Chen T. & Li E. Establishment and maintenance of DNA methylation patterns in mammals. Curr. Top. Microbiol. Immunol. 301, 179–201 (2006). 16570848 10.1007/3-540-31390-7_6

[b32] Gordon C. A., Hartono S. R. & Chédin F. Inactive DNMT3B splice variants modulate *de novo* DNA methylation. PLoS ONE 8, e69486 (2013). 23894490 10.1371/journal.pone.0069486 PMC3716610

[b33] Weisenberger D. J. . Role of the DNA methyltransferase variant DNMT3b3 in DNA methylation. Mol. Cancer Res. 2, 62–72 (2004). 14757847

[b34] Lucifero D. . Coordinate regulation of DNA methyltransferase expression during oogenesis. BMC Dev. Biol. 7, 36 (2007). 17445268 10.1186/1471-213X-7-36 PMC1878483

[b35] Sharma S., De Carvalho D. D., Jeong S., Jones P. A. & Liang G. Nucleosomes containing methylated DNA stabilize DNA methyltransferases 3A/3B and ensure faithful epigenetic inheritance. PLoS Genet. 7, e1001286 (2011). 21304883 10.1371/journal.pgen.1001286 PMC3033376

[b36] Jeong S. . Selective anchoring of DNA methyltransferases 3A and 3B to nucleosomes containing methylated DNA. Mol. Cell. Biol. 29, 5366–5376 (2009). 19620278 10.1128/MCB.00484-09 PMC2747980

[b37] Jair K. W. . *De novo* CpG island methylation in human cancer cells. Cancer Res. 66, 682–692 (2006). 16423997 10.1158/0008-5472.CAN-05-1980

[b38] Kautiainen T. L. & Jones P. A. DNA methyltransferase levels in tumorigenic and nontumorigenic cells in culture. J. Biol. Chem. 261, 1594–1598 (1986). 2418016

[b39] Linhart H. G. . Dnmt3b promotes tumorigenesis in vivo by gene-specific *de novo* methylation and transcriptional silencing. Genes Dev. 21, 3110–3122 (2007). 18056424 10.1101/gad.1594007 PMC2081977

[b40] Bourc'his D., Xu G. L., Lin C. S., Bollman B. & Bestor T. H. Dnmt3L and the establishment of maternal genomic imprints. Science 294, 2536–2539 (2001). 11719692 10.1126/science.1065848

[b41] Ooi S. K. . DNMT3L connects unmethylated lysine 4 of histone H3 to *de novo* methylation of DNA. Nature 448, 714–717 (2007). 17687327 10.1038/nature05987 PMC2650820

[b42] Neri F. . Dnmt3L antagonizes DNA methylation at bivalent promoters and favors DNA methylation at gene bodies in ESCs. Cell 155, 121–134 (2013). 24074865 10.1016/j.cell.2013.08.056

[b43] Guo X. . Structural insight into autoinhibition and histone H3-induced activation of DNMT3A. Nature 517, 640–644 (2015). 25383530 10.1038/nature13899

[b44] Jia D., Jurkowska R. Z., Zhang X., Jeltsch A. & Cheng X. Structure of Dnmt3a bound to Dnmt3L suggests a model for *de novo* DNA methylation. Nature 449, 248–251 (2007). 17713477 10.1038/nature06146 PMC2712830

[b45] Chen Z. X., Mann J. R., Hsieh C. L., Riggs A. D. & Chédin F. Physical and functional interactions between the human DNMT3L protein and members of the *de novo* methyltransferase family. J. Cell. Biochem. 95, 902–917 (2005). 15861382 10.1002/jcb.20447

[b46] Bostick M. . UHRF1 plays a role in maintaining DNA methylation in mammalian cells. Science 317, 1760–1764 (2007). 17673620 10.1126/science.1147939

[b47] Sharif J. . The SRA protein Np95 mediates epigenetic inheritance by recruiting Dnmt1 to methylated DNA. Nature 450, 908–912 (2007). 17994007 10.1038/nature06397

[b48] Choi S. H. . Identification of preferential target sites for human DNA methyltransferases. Nucleic Acids Res. 39, 104–118 (2011). 20841325 10.1093/nar/gkq774 PMC3017615

[b49] Ou C. Y., Kim J. H., Yang C. K. & Stallcup M. R. Requirement of cell cycle and apoptosis regulator 1 for target gene activation by Wnt and beta-catenin and for anchorage-independent growth of human colon carcinoma cells. J. Biol. Chem. 284, 20629–20637 (2009). 19520846 10.1074/jbc.M109.014332 PMC2742827

[b50] De Carvalho D. D. . DNA methylation screening identifies driver epigenetic events of cancer cell survival. Cancer Cell 21, 655–667 (2012). 22624715 10.1016/j.ccr.2012.03.045 PMC3395886

[b51] Pandiyan K. . Functional DNA demethylation is accompanied by chromatin accessibility. Nucleic Acids Res. 41, 3973–3985 (2013). 23408854 10.1093/nar/gkt077 PMC3627572

[b52] Wang K. . MapSplice: accurate mapping of RNA-seq reads for splice junction discovery. Nucleic Acids Res. 38, e178 (2010). 20802226 10.1093/nar/gkq622 PMC2952873

[b53] Chen T., Ueda Y., Xie S. & Li E. A novel Dnmt3a isoform produced from an alternative promoter localizes to euchromatin and its expression correlates with active *de novo* methylation. J. Biol. Chem. 277, 38746–38754 (2002). 12138111 10.1074/jbc.M205312200

[b54] Aapola U. . Isolation and initial characterization of a novel zinc finger gene, DNMT3L, on 21q22.3, related to the cytosine-5-methyltransferase 3 gene family. Genomics 65, 293–298 (2000). 10857753 10.1006/geno.2000.6168

[b55] Lei H. . *De novo* DNA cytosine methyltransferase activities in mouse embryonic stem cells. Development 122, 3195–3205 (1996). 8898232 10.1242/dev.122.10.3195

