# Supplementary material for: DNMT3B isoforms without catalytic activity stimulate gene body methylation as accessory proteins in somatic cells
Source: Nat Commun. 2016 Apr 28;7:11453. doi: 10.1038/ncomms11453 (PMC4853477; doi:10.1038/ncomms11453)
Supplement: Supplementary Information — Supplementary Figures 1-11 and Supplementary Reference [file ncomms11453-s1.pdf]

Supplementary Figure 1

a

b

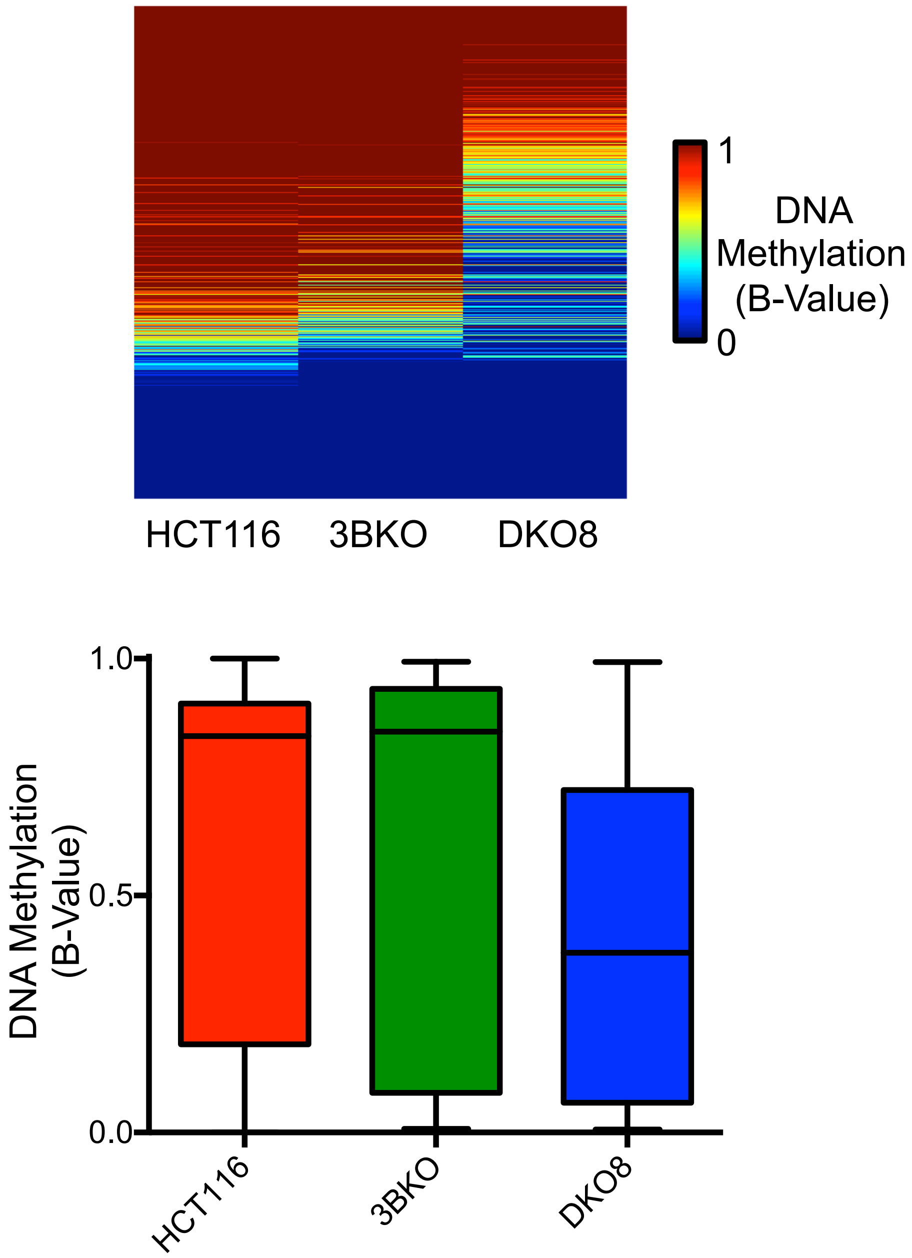

Supplementary Fig. 1. 3BKO and DKO8 cells compared to the parental HCT116 cell line. (**a**) Heatmap showing the 10% (n=39,605) most differentially methylated CpG sites between the 3 cell lines. Each row is an individual CpG site. Color scale ranges from cold to warm (β-value 0-1, 0-100% methylated). (**b**) Boxplot of all CpG sites from the 450K showing DNA methylation distribution of HCT116, 3BKO and DKO8 cell lines.

Supplementary Figure 2

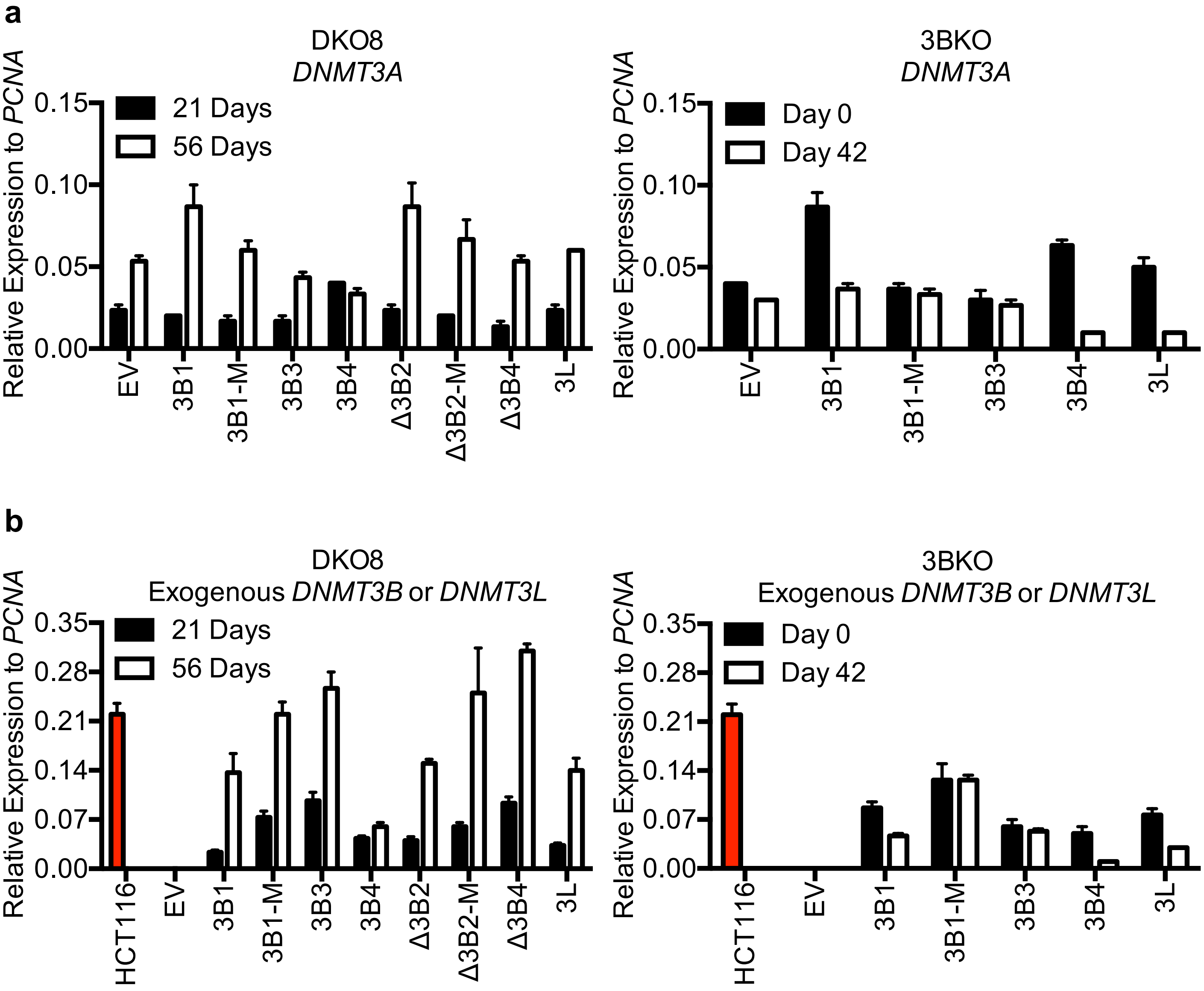

Supplementary Fig. 2. *DNMT3A* and *DNMT3B* mRNA expression levels in DKO8 and 3BKO cells. (**a**) *DNMT3A* mRNA expression levels at days 21 and 56 post transfection in DKO8 cells, expressing exogenous *DNMT3B* or *DNMT3L* normalised to *PCNA* (left). *DNMT3A* mRNA expression levels at days 0 and 42 post 5-Aza-CdR treatment in 3BKO cells expressing exogenous *DNMT3B* or *DNMT3L* normalised to *PCNA* (right). (**b**) mRNA expression level of endogenous *DNMT3B* in HCT116 cells, exogenous *DNMT3B* isoforms and *DNMT3L* at days 21 and 56 post transfection, normalised to *PCNA* in DKO8 cells (left). mRNA expression level of endogenous *DNMT3B* in HCT116 cells, exogenous *DNMT3B* isoforms and *DNMT3L* at days 0 and 42 post 5-Aza-CdR treatment in 3BKO cells, normalised to *PCNA* (right).

Supplementary Figure 3

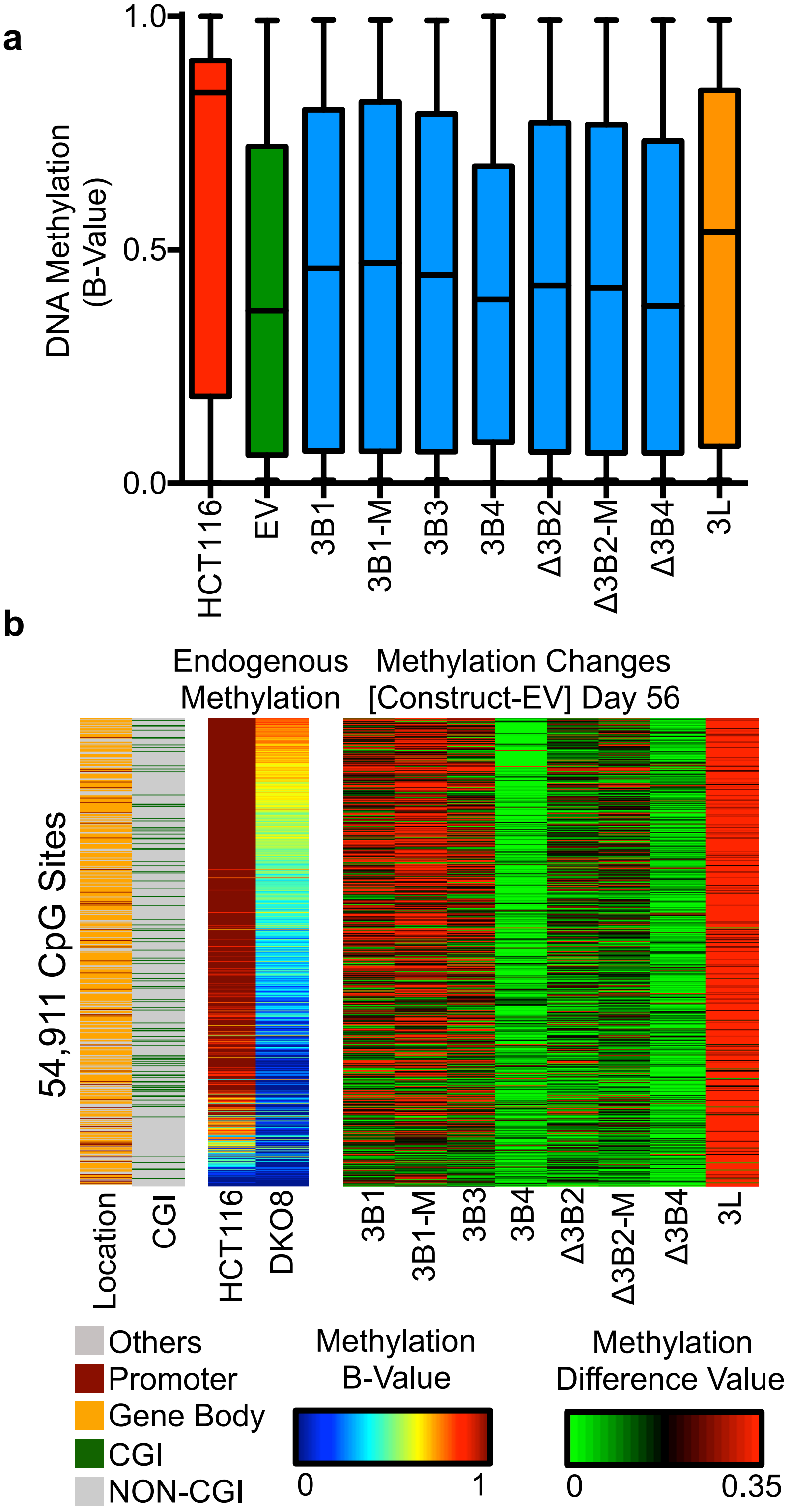

Supplementary Fig. 3. DNMT3B isoforms and DNMT3L restore DNA methylation at specific CpG sites. (**a**) Boxplots showing the distribution of DNA methylation levels of 385,826 CpG sites for each indicated cell line. HCT116 cells global methylation level is included for comparison to the derivative cell line DKO8 EV. (**b**) Heatmap showing 54,911 CpG sites in DKO8 cells, expressing the indicated DNMT isoforms. CpG sites in genomic locations targeted by DNMT3Bs and 3L with respect to promoter (maroon), gene body (orange) and other regions, excluding promoters and gene bodies (gray), are shown in the left panel. CpG sites in CpG islands are represented in green in the left panel. Endogenous methylation levels in HCT116 and DKO8 cells are represented by a cold to warm color scale (β-value 0-1, 0-100% methylated), where every row represents one CpG site in the middle panel. DNA methylation differences between DNMT isoforms and empty vector (EV) are shown in the right panel on a green to red color scale (0=no change).

Supplementary Figure 4

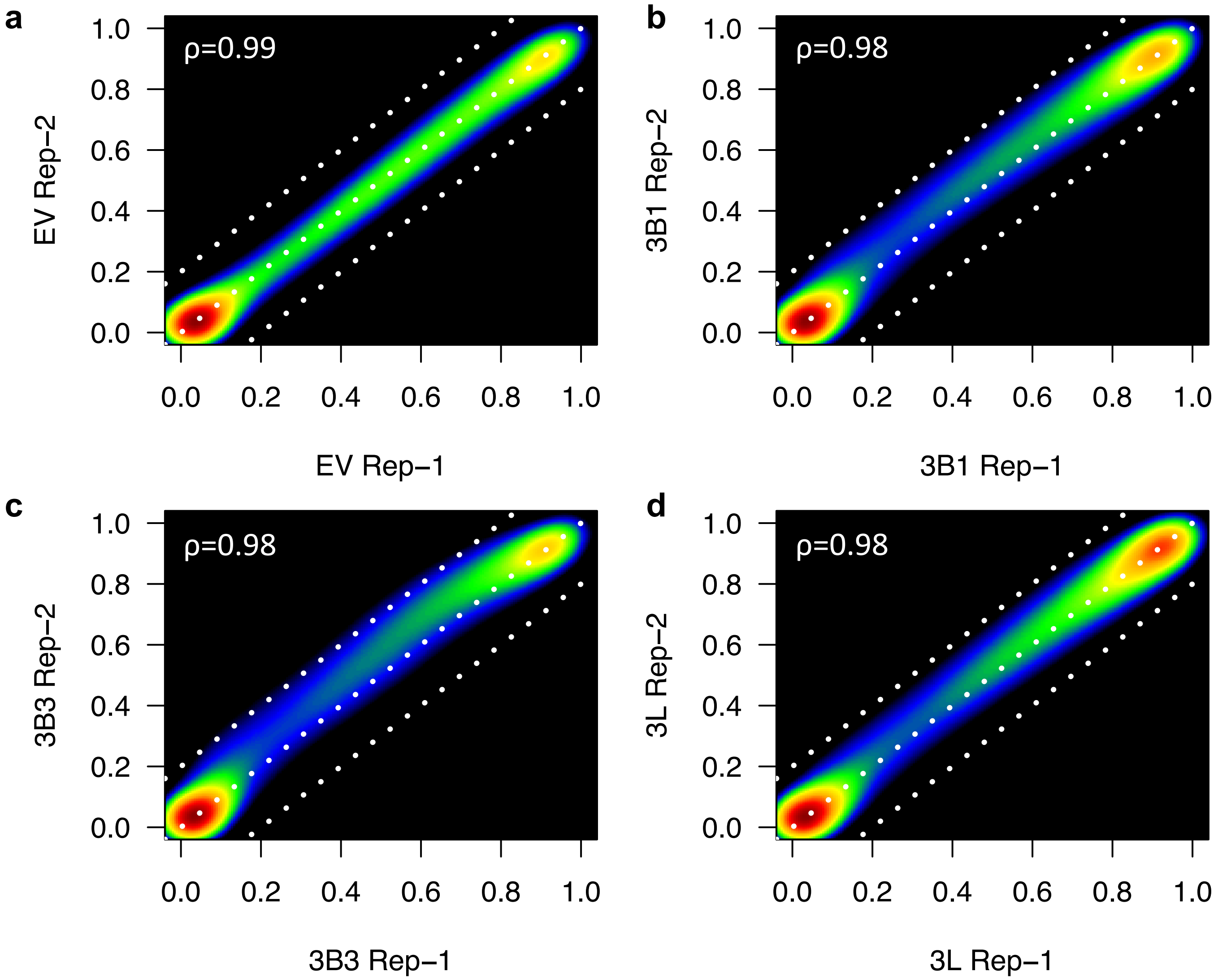

Supplementary Fig. 4. DNA methylation density scatterplots between replicates of DKO8 cells transfected for 56 days with a specific DNMT. Dashed white lines indicate 1:1 line and ± 0.2 Beta-Value differences. Regions of high density are red, low density are blue and black areas represent no density. Spearman correlation values are indicted for each comparison. (**a-d**) Scatterplots of 450K array replicates for the selected constructs EV, DNMT3B1, DNMT3B3 and DNMT3L constructs, respectively.

Supplementary Figure 5

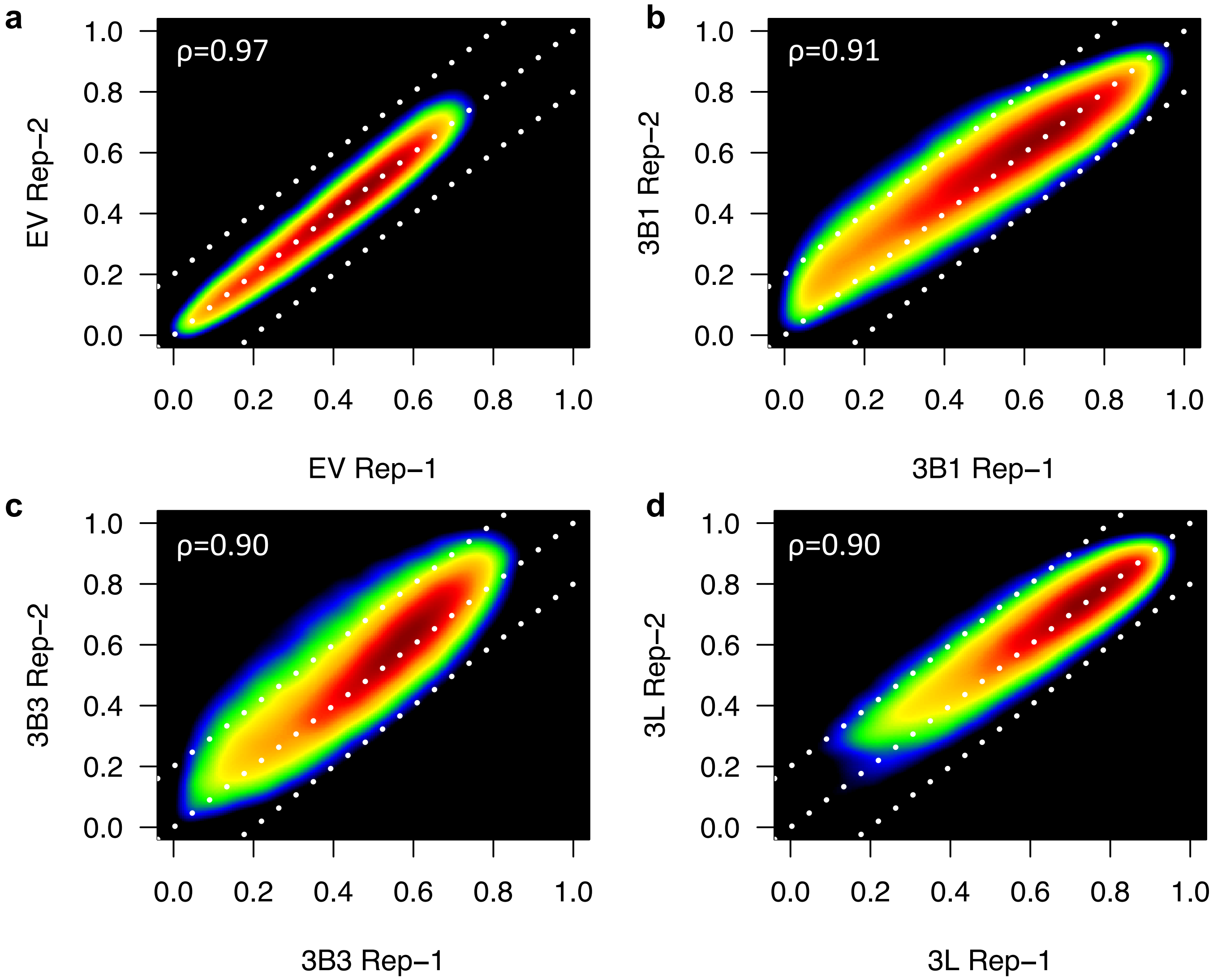

Supplementary Fig. 5. DNA methylation density scatterplots between replicates of DKO8 cells transfected for 56 days with a specific DNMT for targeted CpG sites. Dashed white lines indicate 1:1 line and ± 0.2 Beta-Value differences. Regions of high density are red, low density are blue and black areas represent no density. Spearman correlation values are indicted for each comparison. (**a-d**) Scatterplots of the selected 54,911 CpG sites from the 450K array replicates of EV, DNMT3B1, DNMT3B3 and DNMT3L constructs, respectively.

Supplementary Figure 6

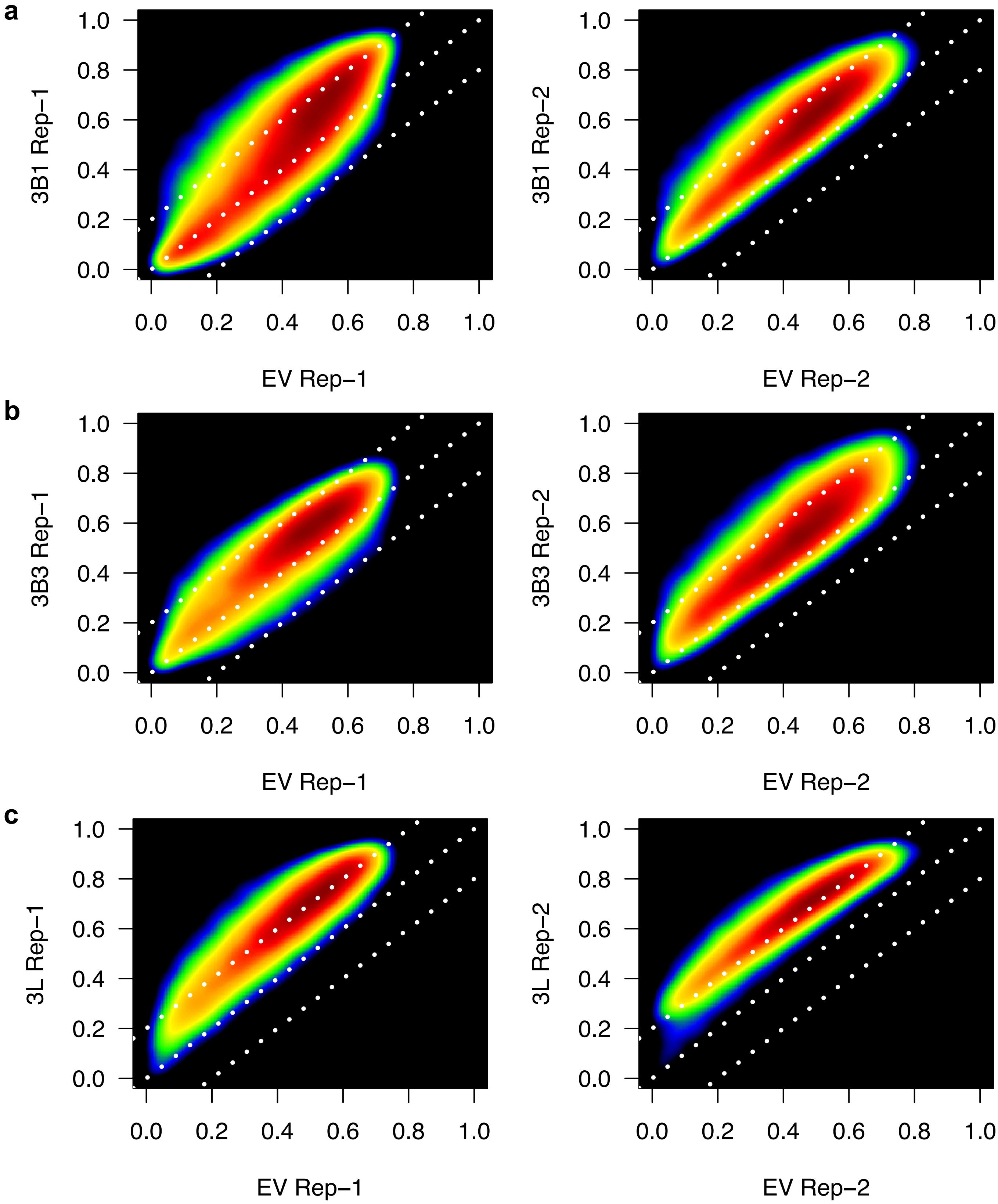

Supplementary Fig. 6. DNA methylation density scatterplots of replicates between EV and transfected cells with a specific DNMT. Dashed white lines indicate 1:1 line and ± 0.2 Beta-Value differences. Regions of high density are red, low density are blue and black areas represent no density. (**a-c**) Scatterplots of the selected 54,911 CpG sites from the 450K array comparing EV to DNMT3B1, DNMT3B3 and DNMT3L with the first array replicate on the left and the second array replicate on the right, respectively.

Supplementary Figure 7

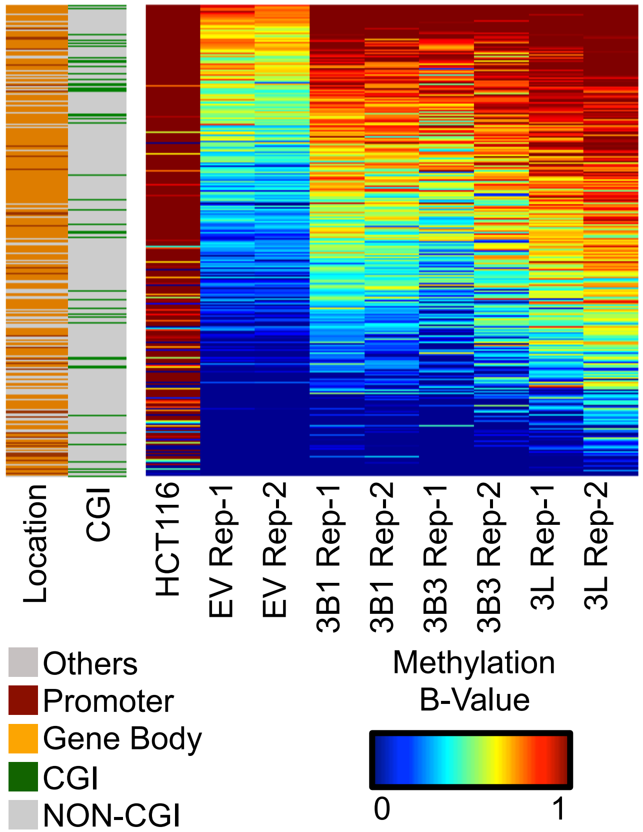

Supplementary Fig. 7. Replicates of DNMT3B isoforms and DNMT3L show consistent target CpG sites. Heatmap showing 54,911 CpG sites in DKO8 cells, expressing the indicated DNMT isoforms. CpGs were selected because they were targeted by at least one construct. CpG sites in genomic locations targeted by DNMT3Bs and 3L with respect to promoter (maroon), gene body (orange) and other regions, excluding promoters and gene bodies (gray), are shown in the left panel. CpG sites in CpG islands are represented in green in the left panel. Endogenous methylation level in HCT116 cells are represented by a cold to warm colour scale (β-value 0-1, 0-100% methylated), where every row represents one CpG. DNA methylation levels of DKO8 cells expressing a DNMT3B isoforms or DNMT3L are shown in the right panel with side by side comparisons of biological array replicates.

Supplementary Figure 8

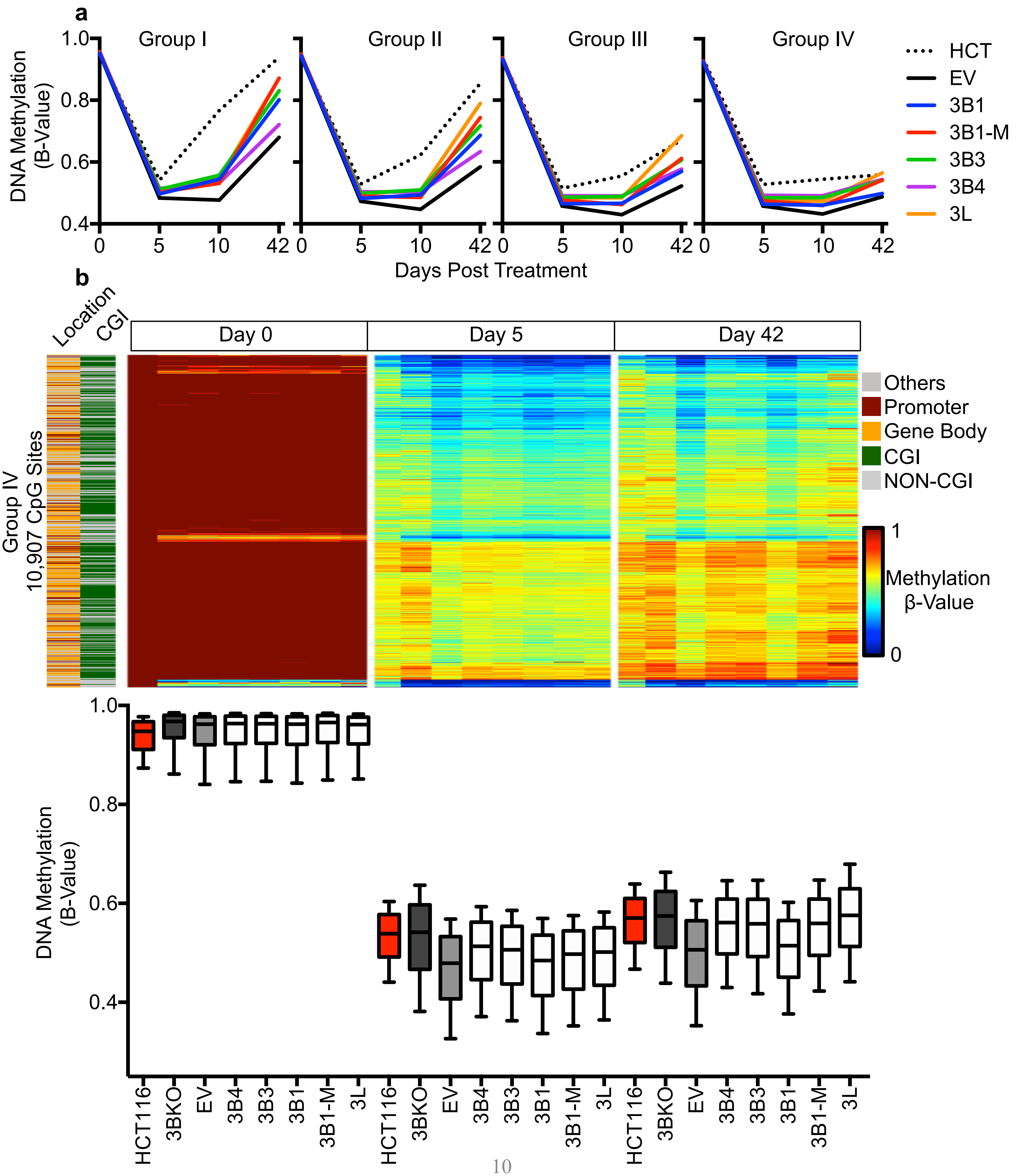

Supplementary Fig. 8. DNMT3B isoforms restore DNA methylation in a DNMT3B knock out cell line. (**a**) Previously described Groups I-IV were used to show average DNA methylation values for each 3BKO cell line expressing a specific DNMT3 isoform at days 0, 5, 10 and 42 post 24 hour 5-Aza-CdR treatment[1]. (**b**) Heatmaps and boxplots showing non H3K36me3-enriched and slow rebounding Group IV CpG sites in cell lines expressing different DNMTs before 5-Aza-CdR treatment and at day 5 and day 42 post treatment[1]. Individual CpG sites falling in genomic locations targeted by DNMT3Bs and 3L in respect to promoter (maroon), gene body (orange) and other regions, excluding promoters and gene bodies (gray) are shown in the left panel. CpG sites in CpG islands are represented in green in the left panel. Endogenous methylation levels in cell lines are represented by a cold to warm color scale (β-value 0-1, 0-100% methylation) where every row represents one CpG site in the 3 right panels.

Supplementary Figure 9

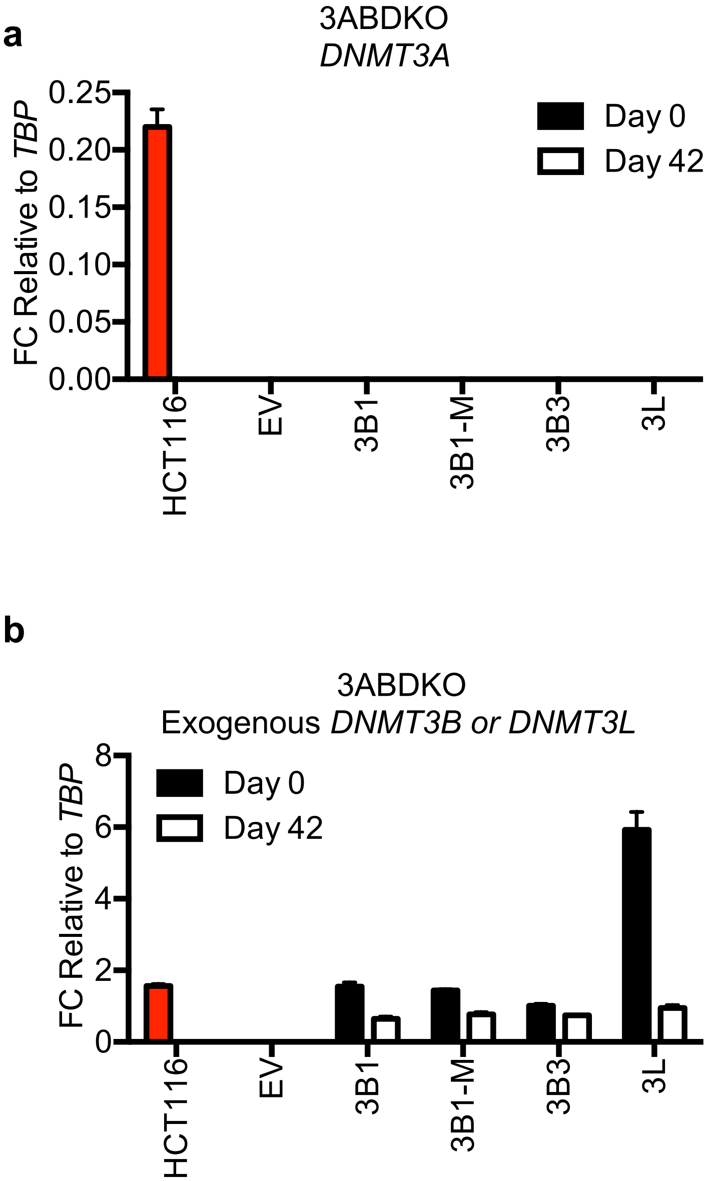

Supplementary Fig. 9. *DNMT3A* and *DNMT3B* mRNA expression levels in 3ABDKO cells. (**a**) mRNA expression level of endogenous *DNMT3A* in HCT116 cells and mRNA expression levels at days 0 and 42 post 5-Aza-CdR treatment in 3ABDKO cells expressing exogenous *DNMT3B* or *DNMT3L,* normalised to *TBP.* (**b**) mRNA expression level of endogenous *DNMT3B* in HCT116 cells, exogenous *DNMT3B* isoforms and *DNMT3L* at days 0 and 42 post 5-Aza-CdR treatment in 3ABDKO cells, normalised to *TBP*.

Supplementary Figure 10

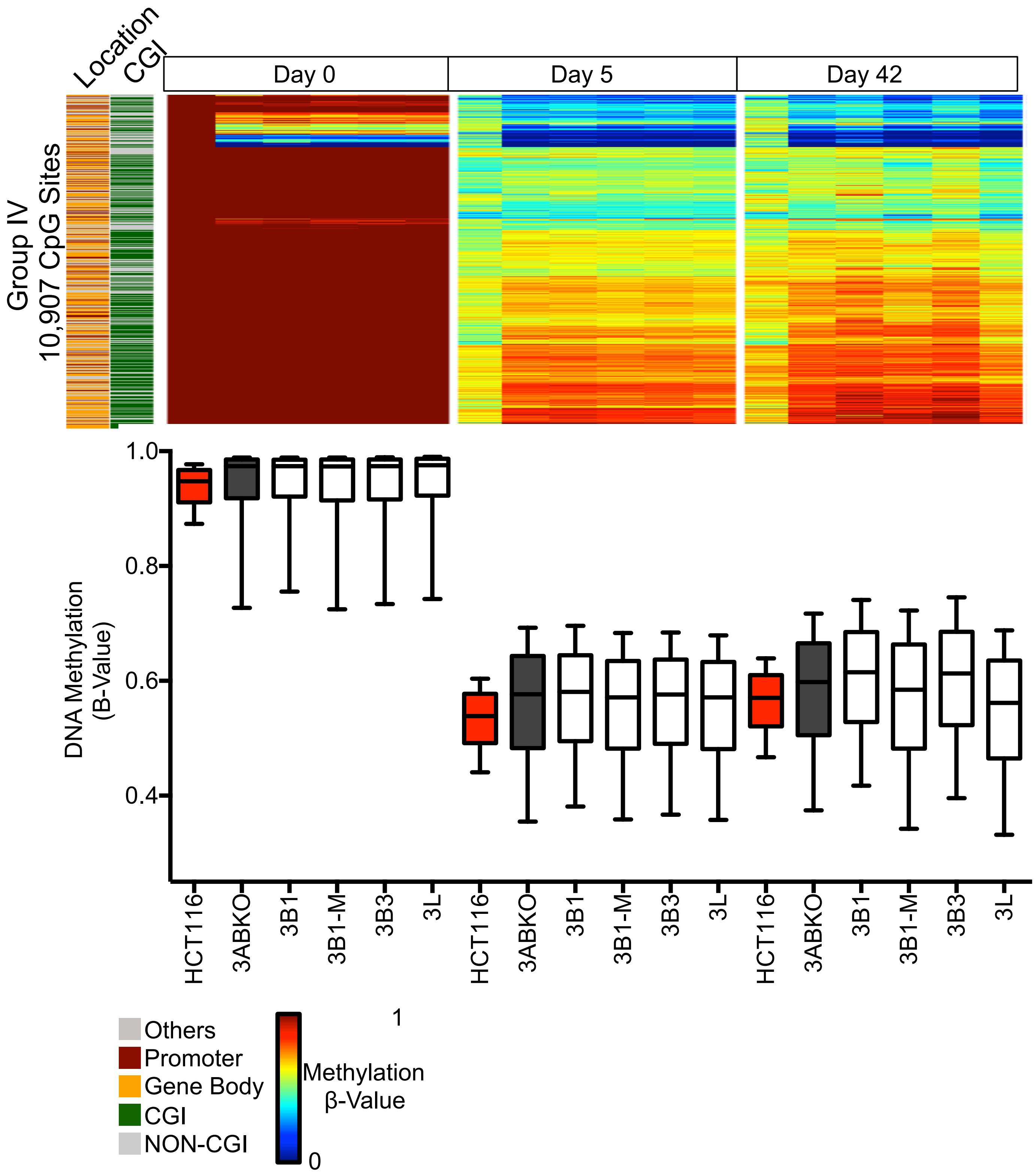

Supplementary Fig. 10. DNMT3B isoforms do not restore DNA methylation in a DNMT3A knock out cell line. Heatmaps and boxplots showing non H3K36me3-enriched and slow rebounding Group IV CpG sites in cell lines expressing different DNMTs before 5-Aza-CdR treatment and at day 5 and day 42 post treatment[1]. Individual CpG sites falling in genomic locations targeted by DNMT3Bs and 3L in respect to promoter (maroon), gene body (orange) and other regions, excluding promoters and gene bodies (gray) are shown in the left panel. CpG sites in CpG islands are represented in green in the left panel. Endogenous methylation levels in cell lines are represented by a cold to warm color scale (β-value 0-1, 0-100% methylation) where every row represents one CpG site in the 3 right panels.

Supplementary Figure 11

Fig 1d.

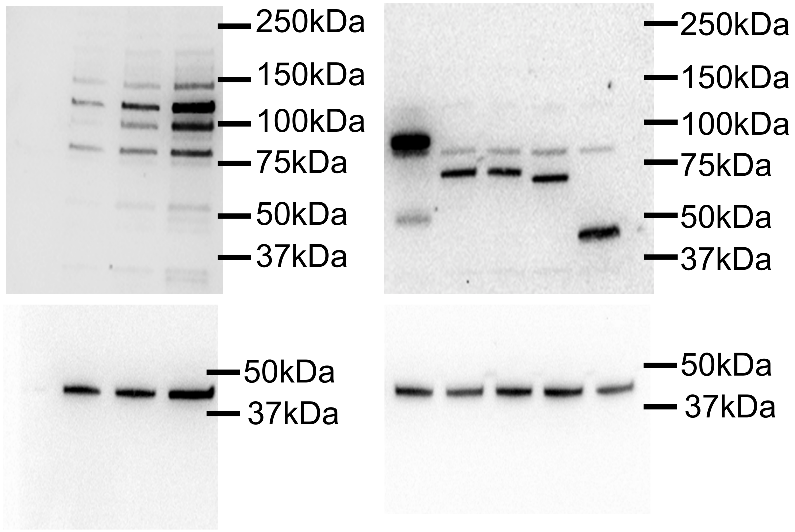

Fig 1e.

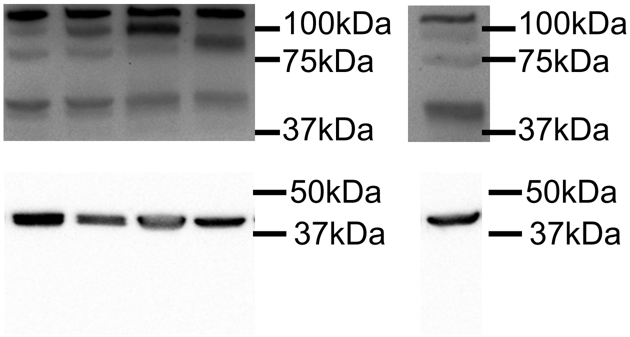

Supplementary Fig. 11. The original full Western blots images used in Figure 1.

Supplementary References

1 Yang, X. *et al.* Gene Body Methylation Can Alter Gene Expression and Is a Therapeutic Target in Cancer. *Cancer Cell* **26**, 1-14, doi:http://dx.doi.org/10.1016/j.ccr.2014.07.028 (2014).
